# Evidence of vascular endothelial dysfunction in Wooden Breast disorder in chickens: Insights through gene expression analysis, ultra-structural evaluation and supervised machine learning methods

**DOI:** 10.1371/journal.pone.0243983

**Published:** 2021-01-04

**Authors:** Behnam Abasht, Michael B. Papah, Jing Qiu

**Affiliations:** 1 Department of Animal and Food Sciences, University of Delaware, Newark, DE, United States of America; 2 Department of Applied Economics and Statistics, University of Delaware, Newark, DE, United States of America; USDA-Agricultural Research Service, UNITED STATES

## Abstract

Several gene expression studies have been previously conducted to characterize molecular basis of Wooden Breast myopathy in commercial broiler chickens. These studies have generally used a limited sample size and relied on a binary disease outcome (unaffected or affected by Wooden Breast), which are appropriate for an initial investigation. However, to identify biomarkers of disease severity and development, it is necessary to use a large number of samples with a varying degree of disease severity. Therefore, in this study, we assayed a relatively large number of samples (n = 96) harvested from the *pectoralis major* muscle of unaffected (U), partially affected (P) and markedly affected (A) chickens. Gene expression analysis was conducted using the nCounter MAX Analysis System and data were analyzed using four different supervised machine-learning methods, including support vector machines (SVM), random forests (RF), elastic net logistic regression (ENET) and Lasso logistic regression (LASSO). The SVM method achieved the highest prediction accuracy for both three-class (U, P and A) and two-class (U and P+A) classifications with 94% prediction accuracy for two-class classification and 85% for three-class classification. The results also identified biomarkers of Wooden Breast severity and development. Additionally, gene expression analysis and ultrastructural evaluations provided evidence of vascular endothelial cell dysfunction in the early pathogenesis of Wooden Breast.

## Introduction

Wooden Breast disease is a muscle disorder of modern commercial broilers that severely impacts meat quality in affected chickens posing a major challenge to the poultry industry. The main phenotypic characteristic of this myopathy is severe hardening of the breast muscle or *pectoralis major* (*p*. *major*). Studies have characterized the major microscopic changes occurring to the affected muscle using histology, which detailed indication of significant multifocal myofiber degeneration and necrosis, inflammation of small caliber veins and venules (phlebitis), and perivascular lipid accumulation in affected birds [1]. Further studies using gene expression, protein and metabolite profiling supported evidence for alterations in calcium homeostasis, hypoxia of the muscle, oxidative stress, fiber-type transition and metabolic dysfunctions, as well as, collagen deposition and cellular repair [2–12]. Although these studies were able to identify various biological processes and pathways associated with the major features of Wooden Breast, the authors maintained that the underlying primary cause of Wooden Breast was unknown.

As described in a previous study [2], Wooden Breast disease is difficult to classify clinically because of the broad range of severity that can be presented from case to case. It is possible that the disease can be subclinical to “developing” in some birds, even when it is clearly clinically evident through palpable muscle firmness and decreased muscle motion in other birds of the same age; this can lead to misclassification as “normal” if subclinical Wooden Breast-affected birds do not exhibit muscle firmness during the time of sampling. While underlying etiology remains to be elucidated, there is demand to develop an accurate, efficient, and objective method for diagnosing and clinically assessing the presence or absence of Wooden Breast disease and its severity from case to case. In a recent study, Praud et al. (2020) used immunohistochemical staining of fibronectin (FN1), neural cell adhesion molecule (NCAM), and myosin heavy chain 15 (MYH15) and provided a quantitative method for examining the extent of fibrosis and regeneration in muscle samples [13]. Another recent study applied supervised machine learning methods to classify chickens as Wooden Breast-affected and unaffected (two-class classification) based on gene expression profiles of breast muscle and liver tissue samples. The authors identified 9 genes enabling correct classification of the chickens in their study [14].

In the current study, we aimed to identify gene expression biomarkers associated with the severity of Wooden Breast. Therefore, in addition to unaffected and severely affected chickens, we included samples from moderately affected chickens to identify gene expression markers for a three-class classification. Using the nCounter MAX Analysis System, we assayed a relatively large number of chickens to allow for a robust examination of four different supervised machine-learning methods. The second aim of our study was to further the understanding of Wooden Breast through biological interpretation of results obtained using various statistical methods. Our findings revealed some novel features of Wooden Breast, discussed in this paper.

## Materials and methods

### Birds

Chickens used in this study were all males obtained from Heritage Breeders (Princess Anne, MD), which were sampled across two genetically distinct purebred lines, B and C, and one commercial crossbred population. The crossbred population was the result of crossing three purebred lines, B, C and D, two of which (lines B and C) were included in this study. The genetic background of these lines was previously described by [15, 16]. Chickens from the purebred lines were raised on the floor, in pens of 300, as explained previously [2]. Crossbred chickens were randomly selected at 29 days of age from commercial broiler farms in the Delmarva region of the United States then transferred to an experimental station for a feed efficacy trial, as described previously [17, 18]. All birds were allowed free access to both feed and water and raised to 7 weeks of age. At 47–48 days of age, birds were euthanized by cervical dislocation and tissue samples were harvested with the Keyes biopsy punch (8 mm; manufactured by HNM) from the caudal edge of the right superficial pectoral muscle perpendicular to myofibers and to the keel bone. Approximately one to two grams of tissue was extracted from each sample location and immediately flash frozen in liquid nitrogen, and stored at -80°C until further processing. The animal protocol used for this scientific study was approved by the University of Delaware Agricultural Animal Care and Use Committee.

### Detection and scoring of disease

The samples for this study were collected when the scoring system on a 5-point scale such as the one developed by Papah et al. (2017) [1] was not available. Therefore, chickens were separated into three classes at necropsy: “unaffected” (U), “partially affected” (P) and “markedly affected” (A). Unaffected chickens showed no apparent gross lesions (i.e., areas of swollen and discolored muscle tissue) and had no detectable increase in firmness of the breast muscle. In partially affected birds, approximately 25–50% of muscle belly was palpably firmer than normal in focally extensive or multifocal pattern. In markedly affected birds, approximately >50% of muscle belly was palpably firmer than normal in widespread multifocal to diffuse pattern. Although White Striping occurrence was not systematically assessed in the current study, all Wooden Breast affected chickens appeared to have some degree of White Striping. The samples used in the current study included 58 unaffected, 21 partially affected and 17 markedly affected birds. Most of the samples were from the commercial crossbred population (n = 73; i.e., 76%), the rest of the samples were from line B (n = 8; i.e., 8%) and line C (n = 15; i.e., 16%). The line B and line C samples, previously used in RNA-seq studies of Wooden Breast [2] and (unpublished study), were included in the current study to increase statistical power.

### RNA isolation and gene expression analysis

Total RNA was isolated from *p*. *major* samples using mirVana™ miRNA Isolation Kit. Quality checks were performed by measuring the concentration and “RNA Integrity Number” of individual RNA samples using NanoDrop 1000 and Agilent Bioanalyzer 2100. The RNA samples were normalized and sent to NanoString Inc. (Seattle, WA, USA) for the quantification of gene expression levels using the nCounter® MAX Analysis System [19]. For this, 192 target genes, along with 12 housekeeping genes (S1 Table) were selected based on multiple RNA-seq experiments conducted in our laboratories [2, 17, 18]. To be cost-effective, we aimed to make this panel useful for multiple projects in our laboratory including a project on Wooden Breast [2] and two projects not directly related to Wooden Breast [17, 18]. However, the majority of the genes in this panel (120 out of 192; i.e., 63%) were of interest in Wooden Breast. Some of these genes have been discussed in previous studies of Wooden Breast [2, 4, 6–9, 11, 20, 21] and their functional analysis using Panther classification system is provided in S2 Table.

### Quality control and normalization of gene expression

Four samples with extremely high binding density were excluded due to saturation effects (NanoString User Manual, 2012). Two other samples were excluded due to an unusual expression pattern, almost the same transcript counts for all genes. Finally, one other sample was previously detected as a duplicate [2] and excluded from the dataset. After excluding these 7 samples, data from 89 birds—16 A, 21 P and 52 U—were used in the subsequent analysis.

For normalization of gene expression, negative control probes were used for detection of “background counts” in each hybridization reaction, and the medians of background counts were applied to background subtraction (NanoString User Manual, 2012). Next, 12 designated housekeeping genes were used for normalization of gene expression (nSolver software package; version 2.5). The raw count data files, normalized transcript counts, and phenotypic data were deposited in the Gene Expression Omnibus (GEO) repository at the National Center for Biotechnology Information (NCBI) (GEO accession number: GSE162634).

### Statistical analysis

As mentioned above, based on gross evaluation of the *p*. *major* muscle at necropsy, chickens were designated as “unaffected” (U), “partially affected” (P) or “markedly affected” (A). We considered this as a three-class classification problem in our analyses. We also considered a two-class classification problem, in which the partially affected and markedly affected chickens constituted one class (A), and the unaffected chickens (U) the other class.

Four statistical learning methods were performed to predict/classify the chickens based on their gene expression levels: Support vector machines (SVM), random forests (RF), elastic net logistic regression (ENET) and Lasso logistic regression (LASSO) [22, 23]. These methods were implemented using the R package caret (6.0–82 version), which is a set of functions that provide a consistent interface to train multiple machine learning algorithms in R [24]. Caret is a short name for Classification And Regression Training (CARET). It was developed for unifying multiple model training and prediction algorithm in R so that it is easy to compare the performance of different methods under the same conditions. For the details of these methods and how we implemented them, please see S1 Text.

### Ultrastructural evaluation

To examine the ultrastructural changes in the vascular endothelium associated with Wooden Breast, we used transmission electron microscopy (TEM) on unaffected and moderately affected *p*. *major* muscle samples derived from previous studies conducted in our laboratory. The unaffected sample was from a crossbred broiler chicken at 2 weeks of age [25] while the moderately affected sample belonged to a chicken from line B at 6 weeks of age [1]. The muscle samples were isolated from the craniolateral aspect of the right *p*. *major* muscle. The muscle tissue samples were then cut into 1 mm 3 pieces and subsequently fixed by immersion into 2% glutaraldehyde and 2% paraformaldehyde in 0.1 M sodium cacodylate buffer at pH 7.4 and stored at 4°C for at least 24 h before further processing. The muscle samples were then processed for TEM at the Delaware Biotechnology Institute (Newark, DE) following the routine protocol described by Papah et al. (2017) [1].

## Results

### Feature selection through supervised machine learning methods

S3 Table presents the results of the four machine learning approaches for both two- and three-class classifications. Out of 192 genes, LASSO, ENET and SVM selected 22, 20 and 14 genes for prediction in three-class classification, and 27, 32 and 11 genes for two-class classification. For LASSO and ENET, the non-zero coefficients for the unaffected, partially affected and markedly affected groups were presented in green, orange and red colors in S3 Table. Recall we used the stepwise method to select a subset of genes for prediction in SVM (see S1 Text). The order in which the gene was added to the final model was reported for SVM in S3 Table. For RF, the ranking of the top 20 genes based on their importance for classification were presented. LASSO and ENET had more common genes than any other methods, which was expected due to their similar method mechanism. SVM had more common genes with LASSO and ENET than with RF, especially for two-class classification. Specifically, for three-class classification, there were five genes selected by three methods: two genes *ARNT2* and *DCTD* were selected by SVM, LASSO and ENET; one gene *TLR2-2* by ENET, SVM, RF; two genes *ZNF650* and *MYH1B* by RF, LASSO and ENET. For two-class classification, two genes *ZNF650* and *MYH1B* were selected by all four methods; five genes *AMIGO2*, *ANKRD2*, *HS3ST2*, *LOC425001* and *NMRAL1* by SVM and LASSO; and one gene *TPI1* by ENET, SVM and FR.

### Performance of supervised learning methods for predicting Wooden Breast status

Tables 1 and 2 report several performance measures of the four supervised learning methods for two- and three-class classifications. Specially, for three-class classification, the power and the false discovery rate were defined separately for markedly affected status and partially affected status. The power to detect markedly affected chickens was defined to be the number of chickens that were correctly identified to be markedly affected divided by the total number of markedly affected chickens. Similarly, the power to detect partially affected chickens was the number of chickens that were correctly identified to be partially affected divided by the total number of partially affected chickens. FDR for the markedly affected chickens was defined to be the number chickens that were falsely predicted as markedly affected, divided by the total number of chickens that were predicted as markedly affected. FDR for partially affected chickens was similarly defined. False positive (FP) for unaffected chickens was defined to be the number of falsely claimed unaffected chicken, which were not unaffected but predicted to be unaffected, divided by the number of unaffected chickens. Accuracy refers to the predication accuracy, the number of accurate predictions divided by the total number of predictions made. See the full confusion matrix in S4 Table.

**Table 1:** Performance measures of the four supervised learning methods for three-class classification.

Method	Power		FDR		FP	Accuracy
	A	P	A	P	U	
LASSO	0.5	0.762	0.333	0.238	0.077	0.809
ENET	0.5	0.714	0.333	0.25	0.077	0.798
SVM	0.562	0.762	0.1	0.158	0.019	0.854
RF	0.562	0.571	0.471	0.333	0.134	0.742

A: markedly affected; P: partially affected; U: unaffected.

FDR: false discovery rate; FP: false positive.

**Table 2:** Performance measures of the four supervised learning methods for two-class classification.

Method	Power	FDR	FP	Accuracy
LASSO	0.838	0.184	0.135	0.854
ENET	0.865	0.180	0.135	0.865
SVM	0.973	0.020	0.077	0.944
RF	0.865	0.238	0.192	0.831

FDR: false discovery rate; FP: false positive.

For this data, SVM achieved the highest prediction accuracy for both three- and two-class classifications with 94% prediction accuracy for two-class classification and 85% for three-class classification.

## Discussion

### Prediction accuracy and genes important for prediction

It is known that no machine learning methods can be uniformly best for all cases and, therefore, it has become a common practice to apply multiple machine learning methods to explore the data and find the one that works best for a specific data [22]. We used four popular supervised learning methods in this paper and found that LASSO and ENET produced the most similar results while SVM selected the fewest predictors with the highest prediction accuracy for both two- and three-class classifications of our data. For three-class classification, LASSO and ENET selected about 20 predictors with around 80% prediction accuracy while SVM selected 14 predictors with 85% prediction accuracy. For two-class classification, LASSO and ENET selected around 30 predictors with about 85% prediction accuracy while SVM selected 11 predictors with 94% prediction accuracy.

We also tried SVM with all 192 predictors. The prediction accuracy was 80% for three-class classification and 87.6% for two-class classification. With the stepwise SVM method, much fewer numbers of genes were needed for higher prediction accuracy. This may indicate that only around 20 genes were really relevant to classification and added genes in the classifier resulted in more noise and led to a reduced prediction accuracy. Therefore, it is important to choose the most relevant predictors in the classifier to improve accuracy.

We used the stepwise approach to RF in a similar way we applied to SVM (see S1 Text). However, since RF randomly selected a subset of predictors for each tree, the predictors included in the RF classifier were not used in each tree and, hence, it was difficult to interpret the contribution of each predictor using the stepwise approach. Therefore, we applied the RF approach using all 192 predictors and reported top 20 genes ranked based on their importance for classification. It turned out that the RF approach had the worst prediction accuracy, 74% for two-class classification and 83% for three-class classification, even compared with SVM with 192 predictors.

Due to the co-expression of genes, the genes that were important for the disease development could be highly correlated with each other. Principal component analysis (PCA) is usually applied to generate principal components, the linear combinations of original predictors that are uncorrelated with each other, and then the machine learning method is applied to the principal components. We tried PCA for both SVM and RF. The radial kernel SVM performed better without PCA while RF performed better with PCA. However, SVM without PCA performed better than RF with PCA. Since it is difficult to interpret the PCA component, we did not consider PCA for further analysis.

In summary, in terms of prediction, SVM was the best choice for this data because it used fewest predictors with the highest prediction accuracy. However, it was not clear how to interpret the influence of each predictor (gene) on the response variable of Wooden Breast status using SVM. On the other hand, LASSO and ENET provided regression coefficient estimates that could be used to interpret how each gene contributes to the Wooden Breast status. For instance, in three-class classification, a positive regression coefficient of a gene for “A” status indicated the increase in expression of the gene would increase the chance of the chicken being markedly affected.

### Biological significance of gene expression results

Gene expression analysis using the nCounter MAX Analysis System and feature selection using various statistical methods including LASSO and ENET revealed specific expression pattern associated with the degree of severity of Wooden Breast myopathy in chickens. Analysis of genes was based on their expression directionality in three categories of the disease severity, namely unaffected, partially affected and markedly affected chickens. For purposes of identifying the genes that are relevant in the progression of Wooden Breast disorder at different stages, we focused on the expression profile of genes with non-zero coefficients (LASSO and ENET) in the partially or markedly affected categories. Under the partially affected category, we examined genes that were either upregulated or downregulated when compared to respective expression in the unaffected and markedly affected categories. Similarly, we examined the genes that were upregulated or downregulated in markedly affected chickens when compared to the expression of the same genes in unaffected and partially affected chickens.

**Fig 1:**
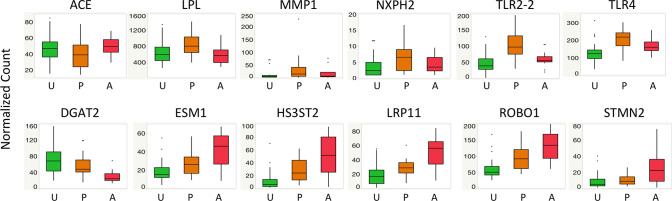
Boxplots of normalized transcript count by Wooden Breast status. Figure illustrates the expression pattern of 12 out of the 20 genes with a non-zero coefficient for the partially affected category (first row) and markedly affected category (second row) in LASSO or ENET analyses. *ACE (angiotensin I converting enzyme); DGAT2 (diacylglycerol O-acyltransferase 2); ESM1 (endothelial cell specific molecule 1); HS3ST2 (heparan sulfate-glucosamine 3-sulfotransferase 2); LPL (lipoprotein lipase); LRP11 (LDL receptor related protein 11); NXPH2 (neurexophilin 2); MMP1 (matrix metallopeptidase 1); ROBO1 (roundabout guidance receptor 1); STMN2 (stathmin 2); TLR2-2 (toll like receptor 2 type 2) TLR4 (toll like receptor 4)*.

Under the partially affected group, 6 genes, *lipoprotein lipase (LPL)*, *matrix metallopeptidase 1 (MMP1)*, *myosin*, *heavy chain 1B (MYH1B)*, *neurexophilin 2 (NXPH2)*, *toll like receptor 2 type 2 (TLR2-2)*, *toll like receptor 4 (TLR4*), were upregulated while 4 genes, *angiotensin I converting enzyme (ACE)*, *adhesion molecule with Ig like domain 2 (AMIGO2)*, *glyceraldehyde-3-phosphate dehydrogenase (GAPDH)*, *NmrA like redox sensor 1 (NMRAL1*), were downregulated when compared to the unaffected and markedly affected categories. Conversely, 8 genes exhibited stage-wise upregulation with the severity of the disorder including *dCMP deaminase (DCTD)*, *endothelial cell specific molecule 1 (ESM1)*, *heparan sulfate-glucosamine 3-sulfotransferase (HS3ST2)*, *LOC427654*, *LDL receptor related protein 11 (LRP11)*, *phosphoglycerate dehydrogenase (PHGDH)*, *roundabout guidance receptor 1 (ROBO1)*, *stathmin 2 (STMN2)*. In this case, the genes showed the highest expression in the markedly affected category, intermediate expression in the partially affected category, and lowest expression in the unaffected category. Additionally, 2 genes, *diacylglycerol O-acyltransferase 2*, *(DGAT2)*, and *chromosome 15 open reading frame 40 (C15orf40)*, exhibited stage-wise downregulation with the disease severity where the expression was highest in the unaffected group, intermediate in the partially affected group and lowest in the markedly affected group. Fig 1 illustrates the expression pattern of 12 out of the 20 genes with a non-zero coefficient (LASSO or ENET) in the partially and markedly affected categories.

### Increased lipid metabolism in partially affected chickens

Analysis of gene expression data using LASSO or ENET revealed *LPL* and 9 other genes with a non-zero coefficient for the partially affected group (S3 Table), suggesting these 10 genes are markers of interest for the diagnosis of moderate Wooden Breast. The upregulation of *LPL* in partially affected chickens compared to both unaffected and markedly affected chickens (Fig 1) suggests an elevated triglyceride hydrolysis activity and uptake of lipids from circulation into the muscle tissue in the former group of chickens. In line with the observation that *LPL* is expressed by the vascular endothelial cells in the *p*. *major* muscles of chickens [20], the findings in the present study suggest a likelihood of a metabolic change in the vascular endothelial cells in partially affected chickens that result in an increase of *LPL* expression. Indeed, a previous study conducted on chickens at 2–3 weeks of age in the early phase of Wooden Breast, showed the upregulation of *LPL* expression in affected chickens in comparison to unaffected ones [7, 8], suggesting early phase of Wooden Breast disorder (at week 2–3 of age) exhibits a similar disease profile as that of partially affected chickens at week 7. Based on this observation, therefore, it is plausible to suggest that endothelial cells of the capillary and venous ends within the *p*. *major* muscles of partially affected chickens undergo a metabolic shift that causes their activation. As manifested by the elevated expression of *LPL*, this endothelial activation then results in increased lipoprotein hydrolysis and uptake of lipids across the vascular wall into the surrounding tissues as observed previously [1, 26, 27].

*Low-density lipoprotein receptor- related protein 11* (*LRP11*), was selected by LASSO and ENET with non-zero coefficient for unaffected and markedly affected groups respectively (S3 Table). Coupled with the stage-wise upregulation of *LRP11* with the severity stages of WB (Fig 1), the current study demonstrates *LRP11* as a potential marker for WB. The protein encoded by *LRP11*, also referred to as *sorting-related receptor with A-type repeats* (*SorLA*), *LR11* or *SORL1* [28], is structurally related to the members of the low-density lipoprotein receptors (LDLRs) gene superfamily [29]. LRP11 protein also constitutes the vacuolar protein sorting 10 protein (Vps10p) domain family of intracellular sorting receptors involved in the intracellular trafficking of proteins from the Golgi network into vacuoles and other organelles [28, 30]. *LRP11* has been implicated in the disruption of lipoprotein metabolism [30, 31], and promotion of atherosclerosis [30]. Similarly, the stage-wise upregulation of *LRP11* with WB-affected chickens in the present study suggests its contribution in the dysregulation of lipid metabolism and vascular damage in the disease process, therefore, affirming our previous findings about the same [7, 8, 20].

In the present study, a gene with a crucial role in triglyceride metabolism, *Diacylglycerol acyltransferase 2* (*DGAT2*), was selected by LASSO with non-zero coefficient in markedly affected group (S3 Table) and exhibited stage-wise downregulation with the WB-severity (Fig 1). *DGAT2* together with *DGAT1* are isoforms of diacylglycerol acyltransferases (DGATs) involved in the catalysis of the final step of triacylglycerols (TAGs) synthesis [32]. TAG synthesis has been demonstrated to reduce cellular lipotoxicity induced by the long chain saturated fatty acids in non-adipose tissues in animals [33]. Along these lines, studies on WB have shown that increased lipid infiltration leading to lipotoxicity contribute to the development of WB in chickens [1, 34]. Based on these observations, the reduced expression of *DGAT2* in WB-affected chickens exhibited in the present study suggests a decreased formation of TAG, and therefore, increasing levels of deleterious fatty acids within the muscle tissue causing further lipotoxicity. As such, it is possible that the suppressed *DGAT2* gene expression indirectly increases severity of WB due to lipotoxicity.

Similar to *LPL* expression, the partially affected group also exhibited upregulation of *MMP1* compared to unaffected and markedly affected groups (Fig 1) and received a non-zero coefficient for this gene in LASSO analysis (S3 Table). MMP1 is known to play key roles in degradation of the extracellular matrix and matrix remodeling, proliferation of endothelial cells, angiogenesis as well as promotion of immune cells in pathologic conditions [35, 36]. Interestingly, *MMP1* expression in the vasculature has been reported to be stimulated by oxidized LDL [37], therefore, linking it with increased lipid metabolism frequently observed in the early stages of Wooden Breast [7, 8, 20]. Accordingly, the upregulation of *LPL* in partially affected chickens, which results in increased fatty acid uptake, may be involved in an indirect stimulation of *MMP1* expression. *MMP1* expression in turn drives the extracellular matrix degradation and remodeling around blood vessels thereby promoting leukocyte adhesion and emigration into the perivascular space, eventually resulting in phlebitis frequently observed in the earlier stages of Wooden Breast [1, 26].

Another important group of genes that were upregulated in the partially affected chickens included Toll-like receptors (*TLR2-2* and *TLR4*; Fig 1). These two genes received a non-zero coefficient for the partially affected group in ENET analysis (S3 Table), indicating their distinct expression in this group of chickens compared to both unaffected and markedly affected chickens. Generally, TLRs, which reside in cell membrane, play a critical role in the innate immunity by recognizing pathogen associated molecular patterns (PAMPS) and damage associated molecular patterns (DAMPS) released from injured tissues thereby promoting inflammation [38]. A study in diabetes showed a role of TLR4 in promotion of lipid metabolism and lipid accumulation through *LPL* expression and diabetes in cardiomyocytes of mice [39]. Similarly, in the current study, there appears to be an association between TLR4 and LPL metabolic activities in partially affected chickens that causes increased lipid uptake across the vasculature leading to lipotoxicity and initiation of inflammation [34].

One of the genes that received a non-zero negative coefficient for the partially affected group in LASSO analysis (S3 Table), i.e., downregulated in this group of chickens, was *ACE* (Fig 1), a component of the renin-angiotensin system (RAS) that is involved in the conversion of the inactive angiotensin I to its active form angiotensin II. Generally, RAS is involved in the homeostatic control of body fluids and cardiovascular functions [40]. Interestingly, *ACE*, which is also expressed by endothelial cells, has also been reported to be linked with bioenergetics control involving carbohydrate and lipid metabolism. In this case, ACE deficiency in mice resulted in decreased abdominal fat deposition, and increased lipid metabolism including upregulation of *LPL* in the liver [41], which partly agrees with the observation in the current study. These observations, therefore, provides more evidence of the role of increased lipid metabolism during the early phase of Wooden Breast myopathy.

### Evidence of vascular endothelial dysfunction

Gene expression analysis of the *p*. *major* muscles from affected chickens revealed evidence of perturbations in the integrity and metabolism of the vascular endothelium that frequently result in endothelial cell dysfunction. This was exemplified by the increased expression of *ESM1*, with the severity of disease (Fig 1). This gene, which received a non-zero coefficient for the markedly affected group in ENET analysis (S3 Table), showed the highest expression level in this group of chickens (Fig 1). *ESM1*, expressed by the vascular endothelium, encodes endocan, a secreted protein often detected systemically. *ESM1* is known to be expressed in activated endothelial cells of blood vessels with pathological disorders such as inflammatory conditions. Consequently, *ESM1* has been considered a biological marker for vascular endothelial inflammation or dysfunction [42, 43]. Therefore, the increased expression of *ESM1* in affected chickens observed in our study suggests existence of an inflammatory insult affecting the vasculature within the *p*. *major* muscles. This observation is consistent with previous studies in Wooden Breast which revealed early onset of vascular involvement in the disease progression as evidenced by artery-sparing lymphocytic phlebitis and perivascular lipid infiltration [1, 26]. Endocan has also been associated with several metabolic disorders in humans such as atherosclerosis [42], acute myocardial infarction [43] and diabetes mellitus [44], which have been known to frequently alter the integrity and metabolic homeostasis of the vascular endothelium resulting in endothelial dysfunction. A disturbance in the integrity and metabolic homeostasis of the vascular endothelium often results in a state of endothelial dysfunction. Under the endothelial dysfunction state, endothelial cells (ECs) switch from their quiescent physiological phenotype to a more biologically active state exhibiting increased metabolic activity [45]. Endothelial cells under a dysfunctional state assume a proinflammatory, pro-adhesive, proapoptotic, proatherogenic and pro-coagulant phenotype [45, 46]. Indeed, this presentation corroborates the molecular observation in our previous study on Wooden Breast that showed increased inflammatory response, cell adhesion and vascular pathology at an earlier stage of the disease [7]. Similarly, microscopic changes of the small-caliber blood vessels were observed in the early phase of Wooden Breast [1]. This, therefore, suggests that the early pathogenesis of Wooden Breast is likely associated with endothelial cell dysfunction, especially in the capillaries and venous ends of the vasculature.

**Fig 2:**
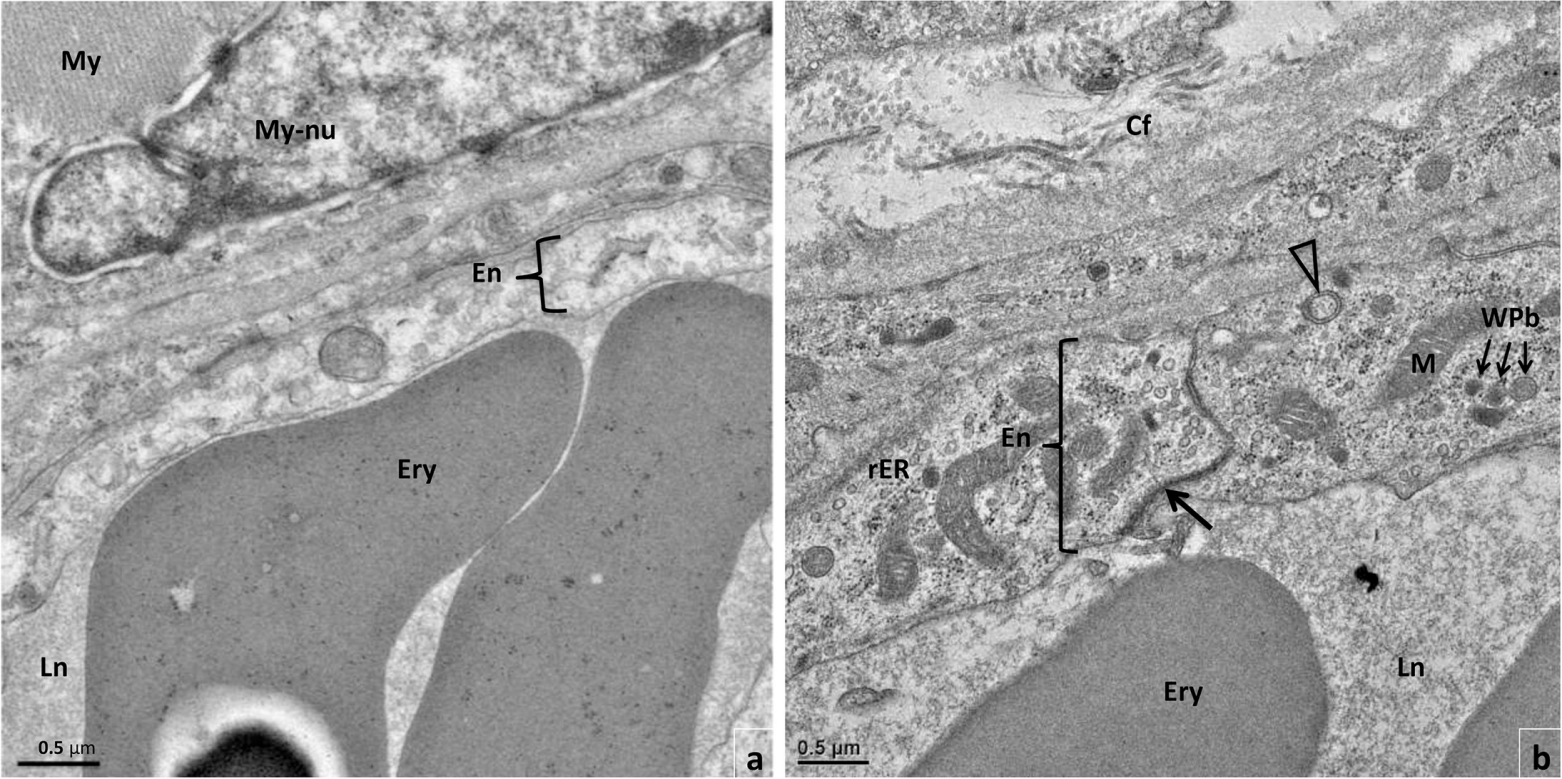
Transmission electron microscopy micrographs of portions of vascular endothelial cell layer (En) from the *pectoralis major* (*p*. *major*) muscle. (a) Unaffected chicken and (b) partially affected chicken. Compared to the relatively thinner endothelial cell layer from the unaffected chicken, the vascular endothelial layer from *p*. *major* muscles of Wooden Breast-affected chicken appears thicker with prominent and numerous organelles including mitochondria (M), ribosomes and rough endoplasmic reticulum (rER), membrane bound multivesicular body (open arrowhead), Weibel-Palade bodies (WPb) and a conspicuous tight junction (arrow). The vascular endothelial cells in the unaffected muscle depict a quiescent phenotype while those in the Wooden Breast-affected muscle presents an activated biosynthetic phenotype. Also shown are the intact myofiber (My) and myofiber nucleus (My-nu) in unaffected muscle, erythrocytes (Ery) in the blood vascular lumen (Ln), and collagen fibers (Cf) in the extracellular matrix indicating fibrosis in Wooden Breast-affected muscle.

Besides the molecular signature, endothelial dysfunction is also presented by changes in the ultrastructure of ECs. At physiological state, quiescent ECs frequently appear thinner, with a smooth luminal profile, indistinct organelles and junctional complexes, and smaller nuclei. However, in a state of endothelial dysfunction, ECs especially in arteries and veins, present thickened profile and prominent nuclei, rough endoplasmic reticula (rER), mitochondria, vesicles and intercellular junctional complexes coupled with increased cytoplasmic processes directed towards the lumen [47]. Interestingly, the endothelial layer of small caliber veins from Wooden Breast-affected chickens, whose samples we derived from our previous study [1], presented similar ultrastructural features, which was consistent with activated ECs. While the endothelial cell layer from the unaffected *p*. *major* muscle exhibited a relatively thinner ultrastructural profile with largely indistinct organelles (Fig 2A), those from the affected *p*. *major* muscle appeared thicker with conspicuous organelles (Fig 2B). Some of the profound ultrastructural changes observed in the endothelial cells of Wooden Breast-affected chickens included prominence of organelles such as mitochondria, rER, ribosomes, junctional complexes and membrane-bound cytoplasmic vesicles including multivesicular bodies (MVB) and Weibel-Palade bodies (WPb) (Fig 2B). Indeed, the ultrastructural presentation of the ECs in *p*. *major* muscle of Wooden Breast-affected chickens depicted a metabolically active and biosynthetic phenotype, suggesting the existence of endothelial cell dysfunction in the face of Wooden Breast disorder.

### Contractile dysfunction

Drastic reduction in postmortem muscle fasciculation has been previously observed in Wooden Breast and attributed to possible damage to either muscle or nerve fibers leading to loss of function within the muscle [2]. Damage to muscle fibers has been documented in numerous studies, but primary nerve damage has not been reported. Speculation about a neurological component of Wooden Breast can further be supported by the results of the current study, as genes that are known to be uniquely or more highly expressed in nervous systems, e.g., *HS3ST2*, *ROBO1* and *STMN2*, were upregulated in Wooden Breast-affected chickens (Fig 1). These three genes received a non-zero coefficient for the markedly affected group, and two of them (*HS3ST2*, *ROBO1)* also received a non-zero coefficient for the unaffected group in ENET analysis (S3 Table), suggesting these two genes are markers of interest for assessing disease severity. Additionally, the gene encoding leucine rich repeat and Immunoglobin-like domain-containing protein 1 (*LINGO1)* which is involved in the modulation of a voltage-gated potassium channel [48], and inhibition of axon regeneration [49] was downregulated in the *p*. *major* muscle of Wooden Breast-affected chickens [7, 8]. This gene has been suggested as a novel therapeutic target in the treatment of CNS diseases [49]. Even as early as 2 weeks of age, the expression of *LINGO1* is downregulated in the chickens that were later diagnosed with Wooden Breast at 7 weeks of age [8]. Another highly relevant gene is *cholinergic receptor*, *muscarinic 4* (*CHRM4*), also known as the *muscarinic acetylcholine receptor M4*, which was upregulated in affected chickens (unpublished data from our laboratory). It has been previously shown that muscarinic acetylcholine receptor M4 is present in neuromuscular junctions and plays a critical role in acetylcholine release in rat muscle [50]. Upregulation of this gene in Wooden Breast-affected chickens may impact elicitation of muscle contraction.

## Conclusions

A critically important aspect of the current study was the inclusion of partially affected chickens, which allowed us to observe changes in gene expression with respect to Wooden Breast severity. For example, when compared with unaffected chickens, partially and markedly affected chickens exhibited higher and unchanged *LPL* expression levels, respectively (Fig 1). This example highlights the importance of including moderately affected chickens when studying the progression of Wooden Breast disease. In fact, if partially affected chickens were not included in the current study, *LPL* and other genes with a similar expression pattern would have gone undetected as relevant genes for wooden breast development.

We used four popular supervised learning methods to analyze the data in this paper and found that SVM was the best choice for this data for prediction purpose because it used fewest predictors with the highest prediction accuracy. However, LASSO and ENET were better methods when it comes to interpreting the contributions of individual genes on the response variable of Wooden Breast status. Therefore, it was important to apply multiple machine learning methods and combine their results together to understand the data better.

The present study expands the findings of previous studies on the involvement of the venous vasculature in the initiation and progression of Wooden Breast in chickens. Using gene expression and ultrastructural evaluations, the present study provides evidence of endothelial cell dysfunction in the early pathogenesis of Wooden Breast in chickens. The occurrence of endothelial cell dysfunction suggests a possible presence of an insult that causes the endothelial cells to assume a metabolically active and biosynthetic phenotype. The nature of the vascular insults perpetuating this change is not apparent; however, as evidenced by increased expression of *LPL* and subsequent increase in lipid metabolism in partially affected chickens, the role of lipids cannot be ruled out. Further research needs to be performed to identify the primary factors promoting endothelial cell dysfunction in Wooden Breast.

## Supporting information

S1 Table Housekeeping and target genes. (XLSX)

S2 Table Classification of genes of interest in Wooden Breast. (XLSX)

S3 Table Genes selected using supervised machine learning methods. (DOCX)

S4 Table Full tables for confusion matrices. (DOCX)

S1 Text Statistical analysis. (DOCX)
