# Supplementary material for: Evidence of vascular endothelial dysfunction in Wooden Breast disorder in chickens: Insights through gene expression analysis, ultra-structural evaluation and supervised machine learning methods
Source: PLoS One. 2021 Jan 4;16(1):e0243983. doi: 10.1371/journal.pone.0243983 (PMC7781381; doi:10.1371/journal.pone.0243983)
Supplement: S3 Table — (DOCX) [file pone.0243983.s003.docx]

Genes selected using supervised machine learning methods: LASSO, Elastic Net (ENET), Support Vector Machines (SVM), Random Forests (RF) analyses

|  | LASSO^1^ | | ENET^1^ | | SVM^2^ | | RF^3^ | |
| --- | --- | --- | --- | --- | --- | --- | --- | --- |
| Gene | 3-class | 2-class | 3-class | 2-class | 3-class | 2-class | 3-class | 2-class |
| ACE | -0.0887 |  |  |  |  |  |  |  |
| ADAM8 |  |  |  |  |  |  |  | 12 |
| AMIGO2 | -0.1858 | 0.4521 | -0.1593 | 0.4308 |  | 5 |  |  |
| ANGPTL5 |  | -0.1031 |  | -0.2142 |  |  |  |  |
| ANKRD2 |  | 0.3722 | 0.0159 | 0.3943 |  | 4 |  |  |
| ARNT2 | -0.1521 | -0.9137 | -0.1373 | -0.6756 | 3 |  |  |  |
| AVD |  |  |  | -0.0123 |  |  |  |  |
| BAG2 |  |  |  |  | 2 |  |  |  |
| BCAN |  |  |  |  | 3 |  |  |  |
| C15orf40 | -0.2262 | 0.1261 |  | 0.2608 |  |  |  |  |
| CAST |  |  |  |  |  |  | 11 | 10 |
| CKMT2 |  |  |  |  |  |  |  | 14 |
| CNTFR |  |  |  | 0.0092 |  |  |  |  |
| COMP |  |  |  | -0.0465 | 2 |  |  |  |
| DCTD | 0.1108 |  | 0.2187 |  | 2 |  |  |  |
| DGAT2 | -0.4922 |  |  |  |  |  |  |  |
| ESM1 |  | -0.1479 | 0.1408 | -0.3119 |  |  |  |  |
| FAM35A |  |  |  |  |  | 3 |  |  |
| GAPDH | -0.8441 |  |  |  |  |  | 1 |  |
| GHR |  |  |  |  |  |  | 7 | 19 |
| GMPS |  |  |  |  |  | 3 |  |  |
| GRIK1 |  | -0.3097 |  | -0.4108 |  |  |  |  |
| HS3ST2 |  | -0.2714 | 0.0750  -0.1364 | -0.2014 | 1 | 3 |  |  |
| HSPB7 |  |  |  |  | 2 |  | 19 |  |
| IL5RA |  | -0.1150 |  | -0.0684 |  |  |  |  |
| KCNC2 |  |  |  |  |  |  | 8 | 7 |
| LGALS3 |  |  |  |  |  |  | 9 | 9 |
| LOC425001 | 0.0810 | 0.6339 | 0.2477 | 0.6183 |  | 4 |  |  |
| LOC427654 | 0.3455 | -0.2176 |  | -0.2083 | 3 |  |  |  |
| LPL | 0.1563 |  |  |  |  |  |  |  |
| LRP11 | -0.0945 |  | 0.0513 | -0.0752 |  |  |  |  |
| LUZP2 | -0.2523 | -0.0571 |  | -0.1896 | 1 |  |  |  |
| METTL21CP1 |  |  |  |  |  |  | 14 | 4 |
| MMP1 | 0.0064 |  |  |  |  |  |  |  |
| MMP11 |  | 0.2850 |  | 0.2976 |  |  |  |  |
| MT.ND3 |  | 0.4365 |  | 0.4225 |  |  |  |  |
| MUSTN1 |  |  |  |  |  |  | 16 |  |
| MXRA8 |  |  |  |  |  |  | 15 | 16 |
| MYBPC1 |  |  |  |  |  |  |  | 17 |
| MYH1A |  | -0.1454 |  | -0.1766 |  |  |  |  |
| MYH1B | -0.4289 | -0.1963 | 0.0528 | -0.2604 |  | 5 | 20 | 13 |
| MYH1C |  | -0.2991 | -0.1682 | -0.3338 | 2 |  |  |  |
| MYH1E |  |  |  |  |  |  | 6 |  |
| MYOZ2 |  |  |  |  |  |  |  | 20 |
| NIM1 |  |  |  |  |  |  | 3 | 1 |
| NMRAL1 |  | 0.4120 | -0.1704 | 0.4633 | 3 | 4 |  |  |
| NXPH2 | -0.1286 | -0.3506 | 0.0997 | -0.4036 |  |  |  |  |
| PGAM1 |  |  |  |  |  |  | 13 | 6 |
| PHGDH | 0.0434  -0.3591 | -0.0098 |  | -0.0869 |  |  |  |  |
| PPARGC1B |  |  |  | 0.0333 |  |  |  |  |
| PPP1R27 | 0.0870 |  | 0.0205 |  |  |  |  |  |
| PPP1R36 |  | 0.0630 |  | 0.0913 |  |  |  |  |
| PROKR2 |  | 0.0414 | 0.0173 | 0.0018 |  |  |  |  |
| PTX3 |  |  |  |  |  |  | 2 | 2 |
| PVALB |  |  |  |  | 1 |  |  |  |
| RHOBTB3 | 0.0274 |  |  |  |  |  | 5 | 5 |
| ROBO1 | 0.0759  -0.0084 |  | 0.1293  -0.0038 |  |  |  |  |  |
| RXRA |  |  |  |  |  |  |  | 15 |
| SLC25A30 |  |  |  |  |  | 3 |  | 18 |
| SMPX |  |  |  |  |  |  | 18 |  |
| SPP1 |  | -0.0402 |  | -0.1255 |  |  |  |  |
| STMN2 |  | -0.3480 | 0.2946 | -0.2858 |  |  |  |  |
| SYBU | -0.0535 |  |  |  |  |  |  |  |
| TLR2.2 |  |  | 0.5415 |  | 1 |  | 17 |  |
| TLR4 |  | -0.0010 | 0.0412 | -0.0844 |  |  |  |  |
| TPI1 |  |  | 0.1325 | 0.2022 |  | 3 | 10 | 8 |
| USH1C | 0.0507 | 0.0977 |  | 0.1545 |  |  |  |  |
| VAT1L |  |  |  |  |  |  | 12 | 11 |
| WISP2 |  |  |  |  | 3 |  |  |  |
| ZNF650 | 0.5858 | 1.0584 | 0.6596 | 0.7622 |  | 1 | 4 | 3 |

^1^The non-zero coefficients for the unaffected, partially affected and markedly affected groups were presented in green, orange and red color, respectively. In 3-class classification, classes are consisted of “unaffected”, “partially affected” and “markedly affected” groups. In 2-class classification, the partially affected and markedly affected chickens constituted one class, and the unaffected chickens constituted the other class.

^2^Numbers represent the order in which the genes were added to the final SVM model.

^3^Numbers represent the importance ranking of the top 20 genes for classification using RF.
