# Supplementary material for: Evidence of vascular endothelial dysfunction in Wooden Breast disorder in chickens: Insights through gene expression analysis, ultra-structural evaluation and supervised machine learning methods
Source: PLoS One. 2021 Jan 4;16(1):e0243983. doi: 10.1371/journal.pone.0243983 (PMC7781381; doi:10.1371/journal.pone.0243983)
Supplement: S4 Table — (DOCX) [file pone.0243983.s004.docx]

Full tables for confusion matrices

(1) Three Classes

Lasso Regression:

| Lasso with three classes |  |  |  |  |
| --- | --- | --- | --- | --- |
|  | Reference |  |  |  |
| Prediction | Affected | Partial | Unaffected | sum |
| Affected | 8 | 1 | 3 | 12 |
| Partial | 4 | 16 | 1 | 21 |
| Unaffected | 4 | 4 | 48 | 56 |
| sum | 16 | 21 | 52 | 89 |
|  |  |  |  |  |
| power | 0.5 | 0.761904762 |  |  |
| FDR | 0.333333333 | 0.238095238 |  |  |
| FP |  |  | 0.0769231 |  |
| Accuracy |  |  |  | 0.808988764 |

Table B.1

Elastic Net Regression:

| Elastic Net with three classes |  |  |  |  |
| --- | --- | --- | --- | --- |
|  | Reference |  |  |  |
| Prediction | Affected | Partial | Unaffected | sum |
| Affected | 8 | 1 | 3 | 12 |
| Partial | 4 | 15 | 1 | 20 |
| Unaffected | 4 | 5 | 48 | 57 |
| sum | 16 | 21 | 52 | 89 |
|  |  |  |  |  |
| power | 0.5 | 0.714 |  |  |
| FDR | 0.333333 | 0.25 |  |  |
| FP |  |  | 0.0769231 |  |
| Accuracy |  |  |  | 0.7978 |

Table B.2

Support Vector Machine:

| SVM 3 classes |  |  |  |  |
| --- | --- | --- | --- | --- |
| Prediction | Affected | Partial | Unaffected | Sum |
| Affected | 9 | 0 | 1 | 10 |
| Partial | 3 | 16 | 0 | 19 |
| Unaffected | 4 | 5 | 51 | 60 |
| Sum | 16 | 21 | 52 | 89 |
|  |  |  |  |  |
| Power | 0.5625 | 0.76190476 |  |  |
| FDR | 0.1 | 0.15789474 |  |  |
| FP |  |  | 0.019230769 |  |
| Accuracy |  |  |  | 0.8539 |

Table B.3

Random Forests:

| RF 3 classes |  |  |  |  |
| --- | --- | --- | --- | --- |
| Prediction | Affected | Partial | Unaffected | Sum |
| Affected | 9 | 3 | 5 | 17 |
| Partial | 4 | 12 | 2 | 18 |
| Unaffected | 3 | 6 | 45 | 54 |
| Sum | 16 | 21 | 52 | 89 |
|  |  |  |  |  |
| Power | 0.5625 | 0.571428571 |  |  |
| FDR | 0.470588235 | 0.333333333 |  |  |
| FP |  |  | 0.134615385 |  |
| Accuracy |  |  |  | 0.7416 |

Table B.4

(2) Two Classes

Lasso Regression:

| Lasso with two classes |  |  |  |
| --- | --- | --- | --- |
|  | Reference |  |  |
| Prediction | A | U | sum |
| A | 31 | 7 | 38 |
| U | 6 | 45 | 51 |
| sum | 37 | 52 | 89 |
|  |  |  |  |
|  |  |  |  |
| power | 0.837837838 |  |  |
| FDR | 0.184210526 |  |  |
| FP |  | 0.134615385 |  |
| Accuracy |  |  | 0.853932584 |

Table B.5

Elastic Net Regression:

| Elastic Net with two classes |  |  |  |
| --- | --- | --- | --- |
|  | Reference |  |  |
| Prediction | A | U | sum |
| A | 32 | 7 | 39 |
| U | 5 | 45 | 50 |
| sum | 37 | 52 | 89 |
| power | 0.8649 |  |  |
| FDR | 0.1795 |  |  |
| FP |  | 0.1346 |  |
| Accuracy |  |  | 0.8652 |

Table B.6

Support Vector Machine:

| SVM 2 classes |  |  |  |
| --- | --- | --- | --- |
| Prediction | Affected | Unaffected |  |
| Affected | 36 | 4 | 40 |
| Unaffected | 1 | 48 | 49 |
| Sum | 37 | 52 | 89 |
|  |  |  |  |
| Power | 0.972972973 |  |  |
| FDR | 0.979591837 |  |  |
| FP |  | 0.076923077 |  |
| Accuracy |  |  | 0.9438 |

Table B.7

Random Forests:

| RF 2 classes |  |  |  |
| --- | --- | --- | --- |
| Prediction | Affected | Unaffected | Sum |
| Affected | 32 | 10 | 42 |
| Unaffected | 5 | 42 | 47 |
| Sum | 37 | 52 | 89 |
|  |  |  |  |
| Power | 0.864864865 |  |  |
| FDR | 0.238095238 |  |  |
| FP |  | 0.192307692 |  |
| Accuracy |  |  | 0.83146 |

Table B.8
