# Supplementary material for: Evidence of vascular endothelial dysfunction in Wooden Breast disorder in chickens: Insights through gene expression analysis, ultra-structural evaluation and supervised machine learning methods
Source: PLoS One. 2021 Jan 4;16(1):e0243983. doi: 10.1371/journal.pone.0243983 (PMC7781381; doi:10.1371/journal.pone.0243983)
Supplement: S1 Text — (DOCX) [file pone.0243983.s005.docx]

## Statistical analysis

Based on gross evaluation of the pectoralis major muscle at necropsy, chickens were designated as “unaffected” (U), “partially affected” (P) or “markedly affected” (A). We considered this as a three-class classification problem in our analyses. We also considered a two-class classification problem, in which the partially affected and markedly affected chickens constituted one class (A), and the unaffected chickens (U) the other class.

Four statistical learning methods were performed to predict/classify the chickens based on their gene expression levels: Support vector machines (SVM), random forests (RF), elastic net logistic regression (ENET) and Lasso logistic regression (LASSO). These methods were implemented using the R package caret (6.0-82 version), which is a set of functions that provide a consistent interface to train multiple machine learning algorithms in R [1]. Caret is a short name for Classification And Regression Training (CARET). It was developed for unifying multiple model training and prediction algorithm in R so that it is easy to compare the performance of different methods under the same conditions.

Support vector machine is a supervised learning method that aims to create an optimal hyperplane in a high-dimensional space to separate data into classes [2]. In SVM, an optimal hyperplane is the one that maximizes the distances to the closest data points from all classes. In other words, it searches for the hyperplane with maximum margins. An important feature of the SVM is the choice of kernels. Different kernels would give different hyperplanes and the most popular kernels are linear kernel and radial kernel. The Radial Kernel SVM was reported in this paper because it is more flexible, allows for nonlinear hyperplanes and produces more accurate predictions. Two important parameters in the radial kernel SVM are the cost parameter and sigma, where the cost parameter is a regularization parameter that balances the trade-off between the misclassification error and the width of the margin. The sigma parameter amplifies the distance between two data points x and x' and decides how local the SVM classifier is. If the sigma is very small, only the x within the certain distance can affect the predicting point. In other words, smaller sigma tends to make a local classifier with smaller bias and larger variance, larger sigma tends to make a much more general classifier with smaller variance but larger bias. Both hyperparameters were tuned by the leave-one-out cross validation to achieve the best prediction accuracy. The ‘svmRadial’ method option was used for the train function in the R caret package, which essentially uses the Kernlab R package.

To rank the genes in accordance with their contribution to classification in SVM, we used stepwise method to select a subset of genes for prediction. We started with one gene in SMV at a time and chose the gene(s) with the highest marginal prediction accuracy calculated through the leave-one-out cross validation. Then we proceeded to add a new gene or remove an existing gene if the prediction accuracy increased. If the prediction accuracy remains the same with and without a gene, we dropped the gene. We stopped adding or removing genes into the SVM when the prediction accuracy failed to increase. All the variables included in the final SVM were considered important for prediction, while their importance ranking is the order in which they were added into the final SVM. Our final radial kernel SVM had 14 predictors with the optimal parameters being cost=10 and sigma=0.001 for the three-classes data set and had 11 predictors with the optimal parameters being cost= 10 and sigma=0.001 for the two-class data set.

Random forests is a supervised learning method that targets to improve a single decision tree, which tends to overfit data with large variance [2]. It operates by constructing a multitude of decision trees and then combining the results of multiple decision trees to obtain a more accurate and stable prediction than a single decision tree. Specifically, RF creates many bootstrapped samples of the original size from the training sample. Then each bootstrapped sample is used together with a random subset of predictors to grow a decision tree. The multiple random trees, which make the random forest, will then be aggregated to make predictions. The parameters in RF are the number of predictors used in each decision tree and the total number of decision trees. Typically, for a classification problem, the number of predictors used in each split for a tree is set to be the square root of the total number of predictors. In our study, the number of genes for each split in each decision tree was 13, which was the square root of total number of genes, and the number of decision trees was set to be 2000 to increase the stability of the results. The ‘rf’ method option in the train function of the R caret package was used for the RF, which essentially uses the randomForest R package. The caret package has a built-in function to calculate the variable importance of each gene for RF. The importance measure is based on how much the prediction accuracy decreases when the variable is excluded. We selected top 20 genes ordered by variable importance for further analysis and making comparison with other methods [1].

LASSO is a regression method that performs both variable selection and regularization to enhance the prediction accuracy and interpretability of the statistical model it produces. Similar to Ridge regression, LASSO regularizes the regression coefficient estimates by shrinking them toward zero to reduce the variance. However, the advantage of LASSO over ridge regression is that it performs variable selection together with regularization. This advantage is due to the use of L1 penalty ($\lambda\sum|\beta_{j}|)$ in LASSO, which has the effect of forcing some of the coefficient estimates to zero while shrinking the others toward zero. The L2 penalty ($\lambda\sum\beta_{j}^{2})$ used by Ridge regression although has the effect of shrinking all the regression coefficients toward zero; it doesn’t set any of the coefficients exactly equal to zero. Therefore, the ridge regression always generates a model with all predictors, which can be difficult to interpret for large p small n problems. On the other hand, LASSO produces sparse models, which only involve a subset of the predictors and are generally much easier to interpret than those produced by ridge regression. The ‘glmnet’ method option was used for the train function in the R caret package, which essentially uses the Kernlab R package. The ‘glmnet’ method requires specification of two parameters: alpha and lambda. For the LASSO approach, we fixed alpha to be one, and only lambda $\lambda$ was tuned by the leave-one- out cross validation to achieve the best prediction accuracy. The optimal lambda was 0.07 for the three-class data set and 0.02 for the two-class data set.

Although LASSO can achieve the goals of variable selection and regularization simultaneously, it has some limitations. For instance, LASSO selects at most n predictors when the number of predictors, p, is much larger than the number of observations, n. Also, if there is a group of highly correlated predictors, LASSO tends to select one variable from a group and ignore the others. ENET [3] overcomes these limits of LASSO by using a linear combination of the L1 and L2 penalties as the penalty function ($\lambda_{1}\sum\left| \beta_{j} \right|+\lambda_{2}\sum\beta_{j}^{2})$. Similar to LASSO, ENET is a regularization and variable selection method. It regularizes the coefficient estimates and select sparse models. But unlike the LASSO method, it allows to select models with more predictors than the number of observations in the large p small n situations. In addition, ENET tends to select or deselect strongly correlated predictors together. The ‘glmnet’ method option was used for the train function in the R caret package to implement ENET, which essentially uses the Kernlab R package. The two parameters used by the ‘glmnet’ method, alpha and lambda, (functions of $\lambda_{1}$and $\lambda_{2}$) were both tuned by the leave-one- out cross validation to achieve the best prediction accuracy for ENET. In our study, the optimal alpha was 0.84 and the optimal lambda was 0.08 for the three-class data set and 0.02 for the two-class data set. Note that when $\lambda_{1}=lambda, \lambda_{2}=0,$ which corresponds to the setting when alpha is set to be 1 in ‘glmnet’ method, the ENET method reduces to the LASSO method.

Note for the two-class classification problem, LASSO and ENET basically apply the regularization to logistic regression model and for the three-class classification problem; the regularization is applied to the multinomial regression. However, the glmnet package uses a log linear model for the logistic/multinomial regression and hence produces one set of regression coefficients for each class, which has different parameterization from the traditional logistic/multinomial models [4]. The data was mean centered and scaled before any machine learning methods were applied.

1. Kuhn M. Building predictive models in R using the caret package. J Stat Softw. 2008;28: 1–26. doi:10.18637/jss.v028.i05
2. James G, Witten D, Hastie T, Tibshirani R. An introduction to statistical learning : with applications in R. 1st ed. New York: Springer-Verlag; 2013. doi:10.1007/978-1-4614-7138-7
3. Zou H, Hastie T. Regularization and variable selection via the elastic net. J R Stat Soc Ser B Stat Methodol. 2005;67: 301–320. doi:10.1111/j.1467-9868.2005.00503.x
4. Friedman J, Hastie T, Tibshirani R. Regularization paths for generalized linear models via coordinate descent. J Stat Softw. 2010;33: 1–22. doi:10.18637/jss.v033.i01
